# Magnetic Proximity Sensor Based on Magnetoelectric Composites and Printed Coils

**DOI:** 10.3390/ma13071729

**Published:** 2020-04-07

**Authors:** Nélson Pereira, Ana Catarina Lima, Vitor Correia, Nikola Peřinka, Senentxu Lanceros-Mendez, Pedro Martins

**Affiliations:** 1 Centre/Department of Physics, Minho University, 4710-057 Braga, Portugal; nmmsp.18@gmail.com (N.P.); anacatari18@gmail.com (A.C.L.); eng.v.correia@gmail.com (V.C.); senentxu.lanceros@bcmaterials.net (S.L.-M.); 2 Algoritmi Center, Minho University, 4800-058 Guimarães, Portugal; 3 INL-International Iberian Nanotechnology Laboratory, 4715-330 Braga, Portugal; 4 BCMaterials, Basque Center for Materials, Applications and Nanostructures, University of the Basque Country Science Park, 48940 Leioa, Spain; nikola.perinka@bcmaterials.net; 5 IKERBASQUE, Basque Foundation for Science, 48013 Bilbao, Spain; 6 IB-S Institute of Science and Innovation for Sustainability, Minho University, 4710-057 Braga, Portugal

**Keywords:** magnetoelectric, polymer-based composites, magnetic sensor, coils, multiferroic

## Abstract

Magnetic sensors are mandatory in a broad range of applications nowadays, being the increasing interest on such sensors mainly driven by the growing demand of materials required by Industry 4.0 and the Internet of Things concept. Optimized power consumption, reliability, flexibility, versatility, lightweight and low-temperature fabrication are some of the technological requirements in which the scientific community is focusing efforts. Aiming to positively respond to those challenges, this work reports magnetic proximity sensors based on magnetoelectric (ME) polyvinylidene fluoride (PVDF)/Metglas composites and an excitation-printed coil. The proposed magnetic proximity sensor shows a maximum resonant ME coefficient (α) of 50.2 Vcm^−1^ Oe^−1^, an AC linear response (R^2^ = 0.997) and a maximum voltage output of 362 mV, which suggests suitability for proximity-sensing applications in the areas of aerospace, automotive, positioning, machine safety, recreation and advertising panels, among others.

## 1. Introduction

Smart—or responsive—materials are defined as materials capable of changing their properties in a controlled and reproducible way, as a response to environmental changes and external stimuli such as stress, moisture, heat, pH, electric or magnetic fields [1,2].

Since the beginning of the new millennium, strong efforts have been dedicated toward developing novel smart and multifunctional materials, and to integrate them into technological applications [1,3]. Such efforts represent a multidisciplinary research field with contributions and implications in the areas of sensors and actuators, energy, mobility, interactivity and biomedical sciences, among others [4]. This interesting research scenario actively promotes the production, optimization and application of innovative materials with tailored or improved functionalities [4]. Those materials include hydrogels, covalent adaptive network materials [5], photomechanical materials [6], shape-memory alloys, electroactive and magnetoactive materials [7], self-cleaning and self-healing materials, among others [1].

Particularly interesting are magnetoactive smart materials [7,8]. Magnetoactive materials have been used for more than two thousand years (202 BC–220 AD), initially for magnetic compasses [9] and nowadays as essential components for motors, generators and electronic devices [10]. Magnetoactive materials have become particularly optimized for the implementation of precision manufacturing tools, magnetic manipulation systems, memory devices, gyrators, filters, actuators and proximity sensors [11,12]. Magnetoactive materials for proximity sensors are very popular, as they can be used for non-contact object detection beyond the normal limits of inductive sensors, offering very long sensing ranges in a small package and are able to detect objects through walls of non-ferrous metals, stainless steel, aluminum, plastic or wood [13,14].

From the different types of magnetoactive materials that can be used for magnetoactive proximity sensors, magnetoelectric (ME) [15,16] composites and related devices represent a growing field over the last decade, due to the magnetic to electric energy conversion capability [17], the magnetic control of polarization and also the possibility of obtaining self-powered devices [18]. These ME composites have emerged as a solution to overcome the limitations of single-phase ME materials, namely, low-temperature coupling and low-ME effect [19,20], allowing innovative functionalities to develop ultra-fast, multifunctional and miniaturized devices [17,21,22].

In contrast to ME single-phase materials where the coupling occurs intrinsically, ME composite materials exhibit a ME response resulting from the mechanical coupling between a piezoelectric phase and a magnetostrictive phase [20,23]. In this way, ME composites can be divided into two major groups, depending on the characteristics of the piezoelectric component: polymer-based ME materials and ceramic-based ME materials [24,25]. Despite their (up to three orders or magnitude) lower ME-voltage response, polymer-based [26,27] ME materials overcome three of the main limitations of piezoelectric-based ME materials: fragility, non-printability and high dielectric losses [28]. In polymer-based ME composite multiferroics, a non-magnetic piezoelectric, such as poly(vinylidenefluoride) (PVDF) and its copolymer poly(vinylidene fluoride-co-trifluoroethylene) (P(VDF-TrFE)), is typically combined with a non-ferroelectric magnetic filler, such as CoFe_2_O_4_ in the case of nanocomposites [19] or Metglas in the case of laminates [29], being the latter the ones in which the highest ME coefficient (1 kVcm^−1^ Oe^−1^) has been reported [28].

Despite the large application potential in different areas, the typical operation of traditional polymer-based ME composites require two applied magnetic fields, a DC (to drive the magnetostriction) and an external AC (to excite the response and enhance resonant excitation) [30] that complicates the design of devices. Thus the development and integration of printed AC coils can represent a milestone in this research field [28].

**Figure 1:**
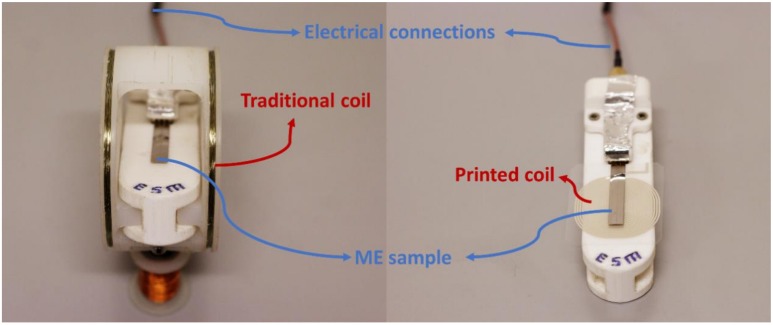
ME sample placed on a traditional coil (left) and on the top a printed coil (right).

In this way, this work reports on the development of a magnetic proximity sensor produced from a polymer-based ME laminate based on Metglas and poly(vinylidenefluoride) (PVDF), combined with a printed magnetic coil (Figure 1).

The selection of the Metglas/PVDF composite is related to the fact that this combination provides the highest ME response and magnetic sensitivity among polymer-based ME materials [31].

## 2. Materials and Methods

Polymer-based ME composites were produced by direct bonding (M-Bond 600 epoxy—Vishay Precision Group, Malvern Pennsylvania, USA, under vacuum of a magnetostrictive alloy of Metglas and a commercial β-PVDF film (Hampton, VA, USA), following the optimized conditions presented in [32]. The 2605SA1 Metglas layer (30 mm × 2 mm × 25 μm, Hitachi Metals Europe GmbH, Düsseldorf, Germany) was magnetized along the length direction (λ = 25 ppm) and the PVDF layer (30 mm × 3 mm × 52 µm) was poled along the thickness direction (d_33_ = −33 pC N^−1^). The coils to be printed were first evaluated by a Finite Element Method Magnetics by an axisymmetric problem analysis, allowing to study the effect of geometry (thickness, spacing and number of turns) in the value of the generated AC magnetic field.

The printed coils were then produced by screen printing, using a semi-automatic screen printer, DX-305D from Shenzhen Dstar (Shenzhen, China), with adjustable speed and with a polyester mesh of 100 wires per centimeter.

The printing process started by printing the silver layer with Metalon HPS-021L from Novacentrix (Austin, Texas, USA) into a polyethylene substrate and cured at 120 °C for 30 min on an electric Convection Oven (JP Selecta 2005165, (Barcelona, Spain). The non-conductive layer was printed with 118-12A/B119-44 solvent-resistant ink from Creative Materials (Ayer, Massachusetts, USA) and cured at 120 °C for 30 min. The process was repeated for the last silver layer achieving an electric bridge from the middle contact.

Optical images of the coil were obtained on a 5M 300x USB Digital Mustech Microscope (Shenzhen, China) with 8 LEDs Brightness Adjustable Measurement Software (MicroCapture Pro.).

The characterization of the printed coil was carried out with a QuadTech 1920 Precision LCR Meter. The inductance (L) and impedance (Z) were obtain in the frequency range 1 kHz to 1 MHz.

The ME characterization of the composite was performed in a system composed of two Helmholtz coils in order to generate an H_DC_ ranging from 0 Oe to 43 Oe, via a DC input current (Keithley 2400, Cleveland, Ohio, USA), being the AC field generated in the printed coil produced with an AC current (Agilent 33220A Function/Arbitrary Waveform Generator, Santa Clara, CA, USA). The ME voltage response was evaluated with a Rigol DS1074Z oscilloscope (Beijing, China).

The voltage ME coefficient (α_33_) was calculated based on Equation (1):

(1) $$\alpha_{33}=\frac{\Delta V}{tH_{AC}}$$

where Δ*V*, *t* and *H_AC_* are the induced ME voltage, the piezoelectric thickness and the *H_AC_* value, respectively.

To validate the use of the ME material (Figure 1) as proximity magnetic sensor (ME composite + printed coil) its voltage response has been studied as a function of the distance to a commercial magnet (KJ Magnetics, Pipersville, PA, USA) and compared with the output value obtained on a Hall sensor (Hirst Magnetic Instruments gm08 Gaussmeter, Falmouth, UK).

## 3. Results and Discussion

**Figure 2:**
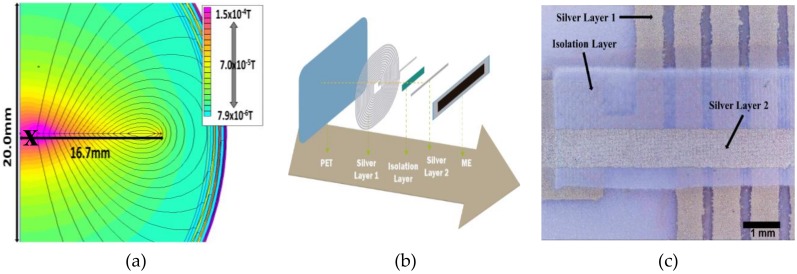
(**a**) Theoretical simulation of the AC magnetic field (in T) generated for a printed coil with a width of 750 µm, 250 µm spacing, 15 turns and a current (I) = 0.02 A. (**b**) Schematic representation of the printing process of the coils. (**c**) Coil printing detail obtained with a digital microscope.

The magnetic coil printing process was performed considering the results obtained through theoretical simulations (Figure 2a). The coil details (width, spacing and turns) were optimized in order to ensure AC magnetic fields in the same order than the ones typically used in polymer-based ME materials (0.1–1 Oe) [31,33].

The color map of Figure 2a reveals that in the region in which the ME composite will be placed (pink-red: X) on a coil with 7 µm thick, 750 µm width, 250 µm lines spacing and 15 turns, AC magnetic fields will be generated in the 0–1.5 Oe range, by varying the electric current from 0 to 0.02 A.

After the printing of the different layers of the coil (PET, silver layer 1, isolation layer, silver layer 2 and ME layer: Figure 2b), the macroscopic quality of the printed material was evaluated by optical images (Figure 2c) that revealed a printed coil with well-defined and compact lines with line width and spacing of ≈750 µm and ≈250 µm, respectively.

**Figure 3:**
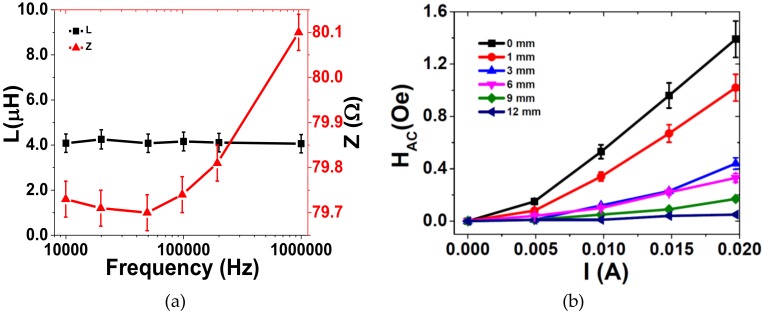
(**a**) Inductance and impedance of the printed coil as a function of frequency. (**b**) H_AC_ value as a function of the distance to the coil and current.

The printed coil’s main features, including inductance and impedance as a function of frequency and H_AC_ generated as a function of the electric current, are presented in Figure 3.

Based on Equations (2) and (3) [34]:

(2) $$X_{L}=2\pi fL$$

(3) $$Z=\sqrt{R^{2}+X_{L}{}^{2}}$$

where *X_L_* is the inductive reactance, f the frequency, *L* the inductance of coil, *Z* the impedance and *R* the resistance, and being the inductance of the coil frequency independence and the *X_L_* value much lower than *R*, the impedance value reported in Figure 3a ≈ 80 Ω is determined by the ink resistivity (10 mΩ/sq- Novacentrix datasheet), line length (1.22 m, obtained from optical images) and printing process (mesh size and curing procedure).

Figure 3b shows an increase in the value of H_AC_ value with increasing electric current (I) and decreasing distance to the coil as represented by Equation (4):

(4) $$H_{AC}=\frac{{µ}_{0}Na^{2}I}{2{\left(a^{2}+z^{2}\right)}^{3/2}}$$

where *H_AC_* is the AC magnetic field generated by the coil, ${µ}_{0}\;$ the vacuum permeability, *N* the number of turns, *a* the radius of the coil in meters, *I* the electric current intensity in amperes and *z* the axial distance in meters from the center of the coil [35].

Based on the previous results, the ME characterization of the sensor was performed on the conditions that promote a higher coupling (higher magnetic AC field: I = 0.02 A and 0 mm distance to the coil).

**Figure 4:**
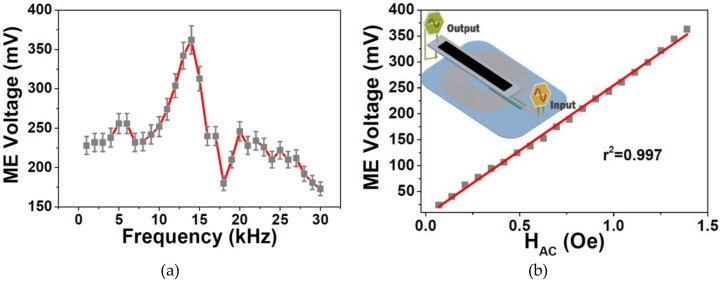
Magnetoelectric (ME) voltage response as a function of: (**a**) frequency and (**b**) H_AC_ magnitude value generated by the printed coil.

For the AC ME characterization, the ME voltage response was studied as a function of the frequency (Figure 4a) and H_AC_ magnitude (Figure 4b).

Figure 4a shows that the ME voltage response strongly increased at ≈13.2 kHz, being reached a maximum voltage value of 362 mV. Figure 4b reveals that the generated voltage increased almost linearly with increasing AC magnetic field up to 1.39 Oe when a maximum voltage of 365 mV was reached. Such high linearity (r^2^ = 0.997) is suitable for the use of these ME composites not only for proximity sensors but also for AC sensing device applications such as digital compasses and earth’s magnetic field sensors [29].

Before the ME composite being tested as a proximity sensor, its ME coupling (α) has been studied as a function of the DC magnetic field intensity (H_DC_).

**Figure 5:**
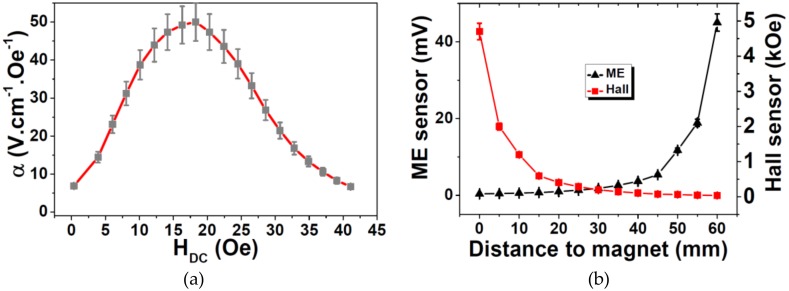
(**a**) ME voltage coefficient (α) response as a function of the H_DC_. (**b**) ME sensor and Hall sensor response as a function of the distance to the magnet.

Figure 5a shows that the ME coefficient (α) increased with increasing applied DC magnetic field up to 18 Oe when a maximum α of 50.2 V cm^−1^ Oe^−1^ mV was reached. Such behavior is explained by the increase of the piezomagnetic coefficient until such optimum magnetostriction field was reached [32]. With further increase of the H_DC_, a decrease of the ME coefficient was achieved, resulting from the saturation of the magnetostrictive effect.

To validate the use of the ME sensor as proximity magnetic sensor (Figure 5b), its response has been studied as a function of the distance to a commercial magnet (KJ Magnetics) and compared with the one obtained on a Hall sensor (Hirst Magnetic Instruments gm08 Gaussmeter, Cornwall, UK). As expected, the Hall sensor’s response decreased with increasing distance to the magnet, being this decrease related with the decrease of the magnetic field. Once the ME sensor increased its response, with increasing H_DC_ for magnetic fields lower than 18 Oe, it was observed an increase in the ME sensor response with increasing distance to the permanent magnet. An obvious consequence of this comparison was that the sensitivity of the ME sensor increased with the distance to the magnet, while the Hall sensor sensitivity decreased in the same conditions.

The developed polymer-based ME proximity sensor, when compared with the three traditional types of proximity sensors [36] (resonant circuit method, bridge method and single-coil method) adds some competitive advantages such as being flexible, versatile, lightweight, low cost, able to conform to complicated shapes obtained from low-temperature fabrication process and foresee the future development of self-power proximity sensors [37].

## 4. Conclusions

A PVDF/Metglas/printed coil proximity sensor device was presented showing suitable characteristics to be applied in sensing applications, particularly in multifunctional flexible devices, due to its good output AC linearity (R^2^ = 0.997), high ME coefficient (50.2 Vcm^−1^ Oe^−1^ at 13.2 kHz and 18 Oe DC field) and large voltage output (362 mV). Other applications such as magnetic transformers, magnetic tools for the automobile/aerospace industry and switches can be based on such ME composite.
